# Leaf Litter and Soil-Mediated Impacts of the Invasive Tree *Prosopis juliflora* on Seedlings of Resident Tree Species

**DOI:** 10.3390/plants15040571

**Published:** 2026-02-11

**Authors:** Dub Isacko Dokata, Simon Kosgey Choge, Pia R. Stettler, Urs Schaffner

**Affiliations:** 1 Rift Valley Eco-Region Research Programme, Kenya Forestry Research Institute, 57, Marigat 30403, Kenya; skchoge2002@gmail.com; 2 Independent Statistician, 3004 Bern, Switzerland; pia.ste@ik.me; 3 Centre for Agriculture and Bioscience International (CABI), Rue des Grillons 1, 2800 Delémont, Switzerland

**Keywords:** allelopathic effects, activated carbon, resident trees, *Neotomia juliflora*, survival, growth

## Abstract

*Prosopis juliflora* is a highly invasive tree species in semi-arid and arid regions in eastern Africa. Its ability to displace herbaceous and woody species has been attributed to allelopathic effects, but this has rarely been tested in competition experiments on natural soil and experimentally binding potentially allelopathic substances. We tested the effect of soil collected underneath and outside of *P. juliflora* canopy, or treated with *P. juliflora* leaf litter, on the survival, growth, and competitive ability of three resident tree species in the presence and absence of activated carbon. Survival and growth of tree seedlings were reduced on soil collected underneath *P. juliflora* canopy and on soil collected outside *P. juliflora* canopy mixed with leaf litter, compared to seedlings growing on soil collected outside *P. juliflora* canopy. When activated carbon was added, seedling performance increased on soil collected underneath *P. juliflora* canopy and particularly on soil collected outside *P. juliflora* canopy mixed with leaf litter. Competition reduced seedling height irrespective of the type of competitor (*P. juliflora* or resident tree species). There was no significant interaction between soil type and competition, indicating that the effect of competition was independent of soil type. The results suggest that *P. juliflora* releases allelochemicals into the soil, which have allelopathic effects on resident tree species, and that at least part of these allelochemicals originate from leaf material.

## 1. Introduction

According to the “novel weapons hypothesis,” some invasive non-native plant species possess novel biochemical weapons that function as powerful allelopathic agents, exerting a direct inhibitory effect on the germination or growth of native plants, or they mediate plant-soil microbial interactions [1,2,3,4]. The inhibitory effect of invasive plants on the growth and development of other plant species may be mediated by organic compounds that are released into the environment as exudates, leachates, or volatiles. Allelochemicals are important mediators in plant-plant interactions if they accumulate in the soil, creating persistent effects on the establishment and growth of interspecific and conspecific plants [5].

The invasive non-native tree *Prosopis juliflora* (Sw.) DC. (Fabaceae), which was recently renamed as *Neltuma juliflora* (Sw.) Raf., is considered to be one of the worst invasive non-native species worldwide [6,7]. It reduces biodiversity and forage for livestock, consumes a lot of water, and increases the densities of disease-transmitting insects [6,8]. Large and dense stands of *P. juliflora* negatively influence plant species richness compared to outside the *P. juliflora* canopy [9]. *Prosopis juliflora* has been found to increase the mortality of native trees, and this effect has been attributed to competition for limited resources such as water or to allelopathy [9,10]. Aqueous extracts from different parts of *P. juliflora*, including the leaves, bark, and roots, exhibit phytotoxic effects on various plant species, including economically significant crops, such as alfalfa, sesame, and sorghum [11]. Studies comparing the inhibitory effects of leaf, bark, and root aqueous extracts on the germination of native grass species found that leaf extracts showed the strongest effect [11,12,13]. The leaf extract and soil solution of *P. juliflora* contains chemicals such as nitriles, alkynes, thiocyanates, carbonyl groups, and alcohols or amines which may contribute to the plant’s allelopathic properties [14].

However, most of the studies on allelopathy of *P. juliflora* published so far were conducted under artificial conditions, e.g., using petri dishes (e.g., [13,15,16,17,18]), artificial potting media, or soil of unspecified origin [7,13,18]. Experiments conducted under artificial conditions may be confounded by experimental artifacts of methodology and thus make it difficult to draw conclusions about the role of allelopathy in natural ecosystems. Also, to our knowledge, methodological approaches to differentiate allelopathy from competition for limiting resources, such as water [19], have not been used in experiments assessing the allelopathic effects of *P. juliflora*. This differentiation is particularly important because the effects of allelopathy can be difficult to separate from other, more pronounced impacts of competition for resources [20,21].

Activated carbon (AC) has been widely used to differentiate between allelopathy and other processes that can influence plant–plant interactions due to its ability to absorb allelochemicals, thereby neutralizing their potential inhibitory effects on plant growth [22,23]. Adding AC to soils can, however, also affect nutrient availability and plant growth directly [24]. Because other techniques to study allelopathy also have their limitations, Lau et al. (2008) [24] suggested that using a set of techniques (e.g., AC, watering with plant extracts, root chamber experiments) provides a robust approach to study allelopathy.

In this study, we assessed the role of allelopathy on the interaction between *P. juliflora* and common tree species in the invaded range in eastern Africa by assessing the impact of soils from underneath and outside the *P. juliflora* canopy and soil mixed with leaf litter on the survival and growth of seedlings in the presence and absence of AC. We hypothesized that (a) in the absence of AC, soil collected underneath *P. juliflora* cover (UPC) reduces the survival, growth, and competitive ability of seedlings from resident tree species; (b) adding leaf litter to soil collected outside the *Prosopis* canopy (OPCLL) also reduces survival, growth, and competitive ability of resident tree seedlings; and (c) the effects of UPC and OPCLL disappear when AC is added to the soil. In our experiments, we used three tree species (*Vachellia tortilis* (Forssk.) *Galasso & Banfi*, *Balanites aegyptica* (L.) *Delile*, and *Ziziphus mauritania* Lam.), which are characteristic of habitats invaded by *P. juliflora* in eastern Africa. Both *V. tortilis* and *B. aegyptica* are native to eastern Africa, while *Z. mauritania* is native to Asia but is widely naturalized in eastern Africa.

## 2. Results and Discussion

### 2.1. Effect of Soil Origin and Activated Carbon on Seedling Survival

**Figure 1:**
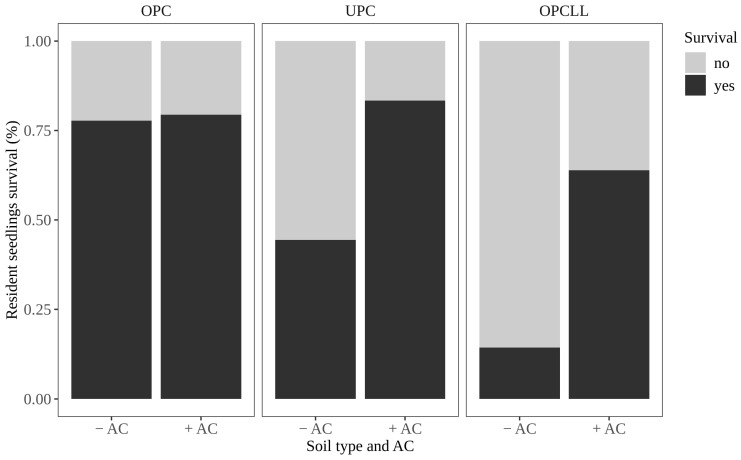
The interacting effect of soil type and activated carbon (AC) on the survival of seedlings of resident tree species. OPC = soil from outside *P. juliflora* canopy; UPC = soil from underneath *P. juliflora* canopy; OPCLL = soil from outside *P. juliflora* canopy mixed with *P. juliflora* leaf litter.

The survival of resident seedlings differed depending on the soil type and presence /absence of AC. In the absence of AC, survival of resident seedlings was 78% in OPC soil but only 44% in UPC and 14% in OPCLL soils (Figure 1). When AC was added, survival of resident seedlings was 83% in UPC and 79% in OPC soils and somewhat lower in OPCLL soils (64%). In particular, adding AC to soil collected outside the *P. juliflora* canopy mixed with leaf litter significantly increased the survival of resident seedlings (Figure 1 and Figure S1a,b; Table S1).

### 2.2. Effect of Soil Type and AC on Resident Seedlings Height

**Figure 2:**
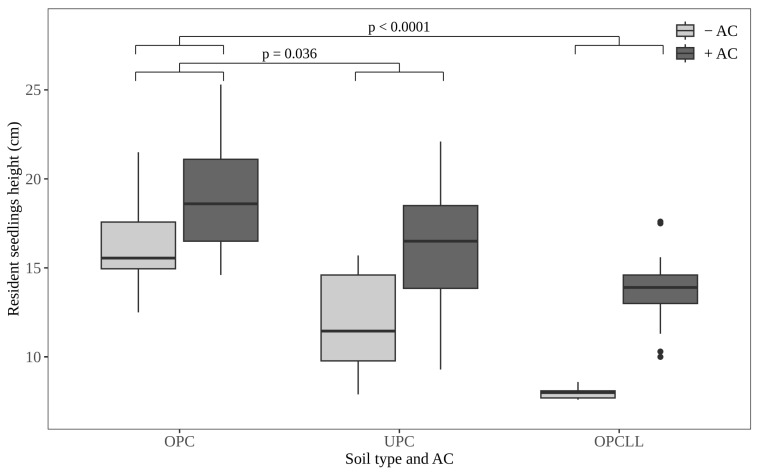
Effect of soil type and activated carbon (AC) on seedling height of resident tree species. OPC = soil from outside *P. juliflora* canopy; UPC = soil from underneath *P. juliflora* canopy; OPCLL = soil from outside *P. juliflora* canopy mixed with *P. juliflora* leaf litter.

The height of resident seedlings was significantly influenced by soil type and by the presence/absence of AC (Figure 2). In the absence of AC, resident seedling height was highest in OPC soils, followed by UPC and OPCLL soil types. With the addition of AC, the increase in seedling height was significantly larger in OPCLL and UPC soils, relative to OPC soil (Figure 2; Table S2).

### 2.3. Effect of Soil Type and Competition on Seedling Height

**Figure 3:**
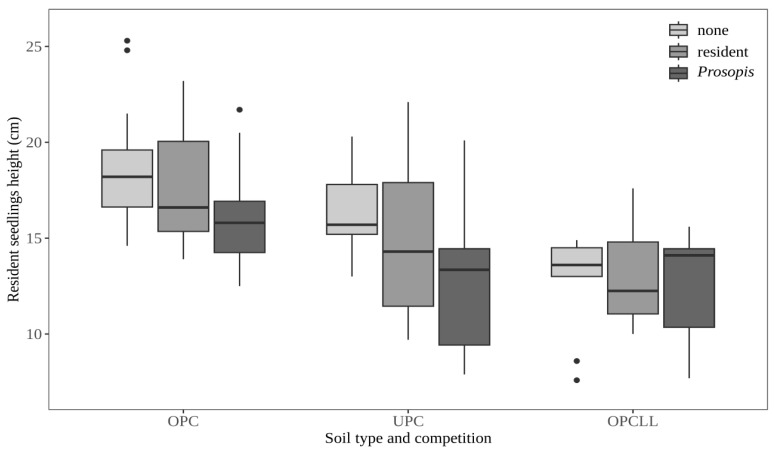
Effect of soil type and competition on seedling height of resident tree species. OPC = soil from outside *P. juliflora* canopy; UPC = soil from underneath *P. juliflora* canopy; OPCLL = soil from outside *P. juliflora* canopy mixed with *P. juliflora* leaf litter.

Competition significantly reduced resident seedling height irrespective of the type of competitor (*P. juliflora* or resident seedlings) (Figure 3; Table S2). There was no significant interaction between the soil type and competition, suggesting that the effect of resident or *P. julifora* competitors was independent of soil type (Table S2). Overall, the lowest seedling height was found in OPCLL soil, followed by UPC and OPC soils, in both the presence and absence of competitor species (Figure 3 and Figure S2a,b).

### 2.4. Effect of Competition and Activated Carbon (AC) on Seedling Height

**Figure 4:**
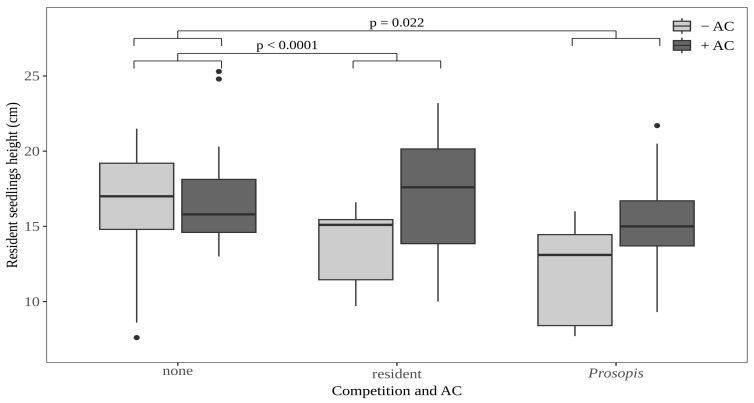
The interacting effect of competition and activated carbon (AC) on seedling height of resident tree species. None = resident seedlings were grown without competitors; resident = seedlings of resident tree species were grown in competition with a seedling of a different resident tree species; *Prosopis* = seedlings of resident tree species were grown in competition with a seedling of *P. juliflora*.

The effect of AC differs between different competition treatments. While there was no direct effect of AC on seedling growth in the absence of competition (Wilcoxon rank sum test: resident tree species: *p*-value = 0.7526, *Prosopis*: *p*-value = 0.8182), the addition of AC increased seedling growth in the presence of both resident and *P. juliflora* seedlings (Figure 4; Table S2).

## 3. Discussion

Our results provide evidence that soil collected from underneath the *P. juliflora* canopy and leaf litter added to soil collected outside the *P. juliflora* canopy incur allelopathic effects on seedlings of resident tree species. Seedlings grown on UPC and OPCLL soils suffered increased mortality, relative to seedlings grown on OPC soil. Similarly, the growth of the resident seedlings was suppressed in UPC and OPCLL soils. When AC was added to UPC and OPCLL soils, the survival and growth of the resident seedlings increased significantly. This suggests that *P. juliflora* releases allelochemicals into the soil, which negatively affect resident trees, and that at least part of these allelochemicals originate from leaf material.

### 3.1. The Effect of Soil Origin

We found that seedling survival and growth of resident tree species differed between soil collected from underneath the *P. juliflora* canopy and soil collected outside the *P. juliflora* canopy. These results are consistent with a study on the impact of the invasive tree *Acacia pennatula* (Benth) on the performance of co-occurring tree species, which revealed suppressed growth of tree seedlings underneath the *A. pennatula* canopy [25]. Similarly, a study on the allelopathy of an invasive tamarisk species (*Tamarix ramosissima* Ledeb., *T. chinensis* Lour.) revealed negative effects of tamarisk on co-occurring plants due to allelopathic effects mediated by the soil in which tamarisk grows [26]. The low survival and stunted growth of resident tree species grown in UPC soils may be explained by the release of toxic plant metabolites that directly inhibit growth [27]. Alternatively, this effect may also stem from the accumulation of soil microorganisms, which render the living conditions unfavorable for seedlings of con- or heterospecific species. For example, Packer and Clay [28] found that sterilization of soil collected underneath trees improved seedling survival relative to unsterilized soil, whereas sterilization of distant soil did not affect survival; the inoculation of healthy seedlings with isolates obtained from dying seedlings reduced seedling growth by 65%.

### 3.2. Leaf Litter as a Source of Allelopathic Substances

Various parts of invasive non-native plants, including leaves, roots, and bark, have been assessed as potential sources of allelopathic substances [17,29]. Overall, leaf litter and root exudates appear to be the main sources of allelochemicals, while bark contributes relatively little to the allelopathic effects of invasive non-native plant species [27,28,29,30]. A previous study [31] indicated that leaves of invasive species are the main source of phytotoxic compounds due to fast decomposition, which enhances the release of allelochemicals. This notion is in line with our findings that adding *P. juliflora* leaf litter to soil caused a strong allelopathic effect on seedlings of resident tree species. Similarly, Parepa et al. [32] found that leaf litter of invasive knotweeds elicited more inhibitory effects on seed germination of native plant species than soil collected underneath the invader’s canopy.

The more pronounced inhibitory effects of soil mixed with leaf litter compared to soil collected underneath *P. juliflora* canopy may be due to a greater concentration of active compounds released from decomposing leaf material, or the chemical composition differs between leaves and roots [22]. Alternatively, exudates of *P. juliflora* may degrade relatively rapidly in soil, thus leaving only a short-term legacy effect in the absence of a continuous influx of plant material [32]. 

In studies assessing the allelopathic effects of leaves of invasive non-native plant species, either leaf litter aqueous extracts or leaf litter fragments were mixed with soil. A study by Huang [33] compared the two treatments when testing the allelopathic effects of *Cinnamomum septentrionale* Hand. Mazz leaves on tree saplings. They found that both treatments negatively influenced the growth, chlorophyll synthesis and photosynthesis of *Eucalyptus grandis* Hill ex Maid saplings, but that leaf litter aqueous extracts had a slightly stronger effect than leaf litter fragments.

### 3.3. Assessing Allelopathy of P. juliflora Using Multiple Techniques Under Semi-Natural Conditions

We combined three techniques to assess the allelopathic effects of *P. julilfora*: the use of soil with a different *P. juliflora* history, the use of AC, and the competitive interactions between resident plants and *P. juliflora.* This was done deliberately to reduce the risk of methodological artifacts in experimental designs as suggested in previous allelopathy studies [17]. Applying a set of techniques and comparing the results provides compelling evidence for the role of allelopathy in species interactions. In particular, we found a significant increase in the height of resident species grown in competition with seedlings of another resident tree species or with *P. juliflora* when AC was added to UPC and OPCLL soils. In contrast, adding AC to OPC soil did not affect the performance of resident seedlings. The positive effect of AC seedling height of resident seedlings grown in competition with *P. juliflora* in the presence of AC can be attributed to the ability of AC to bind allelopathic substances exuded by *P. juliflora* [34,35], thereby mitigating direct inhibitory effects of *P. juliflora* on plant growth or indirect effects mediated by plant-microbe interactions [25,36]. The height of resident seedlings grown in competition with a second resident seedling also increased when AC was mixed into the soil. The mechanisms underlying the positive effect of AC on the height of seedlings grown in competition may differ between the soil types. While seedlings grown on UPC or OPCLL could suffer from soil legacy effects or from allelopathic substances exuded by competing seedlings or leaf litter, resident seedlings grown on OPC are likely to experience allelopathic effects from competing seedlings only. The height increase of resident seedlings competing on OPC soil in the presence of AC can be explained by the allelopathic properties of at least some of the test plant species. In particular, *Vachellia tortilis* might have influenced the results of the experiment as aqueous extracts of different plant parts have been shown to exhibit allelopathic effects on seedlings of crop plants [37].

At the time the soil samples were collected in the field, leaf litter was lying on the ground underneath the *P. juliflora* canopy. However, we did not quantify the amount of leaf litter. It remains to be shown whether the more pronounced inhibitory effect in the OPCLL treatment can be attributed to more allelochemicals released by leaf litter.

## 4. Materials and Methods

### 4.1. Plant and Soil Samples

The seeds of the three resident tree species (*V. tortilis*, *B. aegyptica,* and *Z. mauritania*) and *P. juliflora* were collected from locally available trees one month before the experiment. The seeds of *V. tortilis* and *B. aegyptica* were soaked in warm water for 24 h before sowing to improve germination rates. Immediately after the treatment, the seeds of the two species and of *Z. mauritania* were sown in seed beds prepared at the KEFRI tree nursery. Four days after sowing the seeds of the three resident species, seeds of *P. juliflora* were also sown in the same nursery. This procedure made it possible to obtain seedlings for all test species at the same time. Seeds were watered twice every day, in the morning and in the evening. Weeds that sprouted in the potting tubes containing seedlings of the test species were removed continuously.

Potting media used for raising the seedlings were sourced from KEFRI Marigat Centre in Baringo County, Kenya, either underneath *P. juliflora* canopy (UPC) or outside *P. juliflora* canopy (OPC) from more than 40 *P. juliflora* trees of different sizes. A total of 70 soil cores (20 cm in depth, 5 cm in diameter) each were collected underneath and outside the canopy of *P. juliflora.* The soil cores were pooled and mixed to produce two soil bulk samples. The soil samples were collected a day before setting up the experiment.

Fully developed leaves of *P. juliflora* of 3–10 cm length were collected a fortnight before the experiment was set up. The leaves were cleaned with fresh water, sun-dried, crushed, and blended to form a fine powder [17]. The powdered leaf samples were stored in darkness at room temperature. On the day the experiment was set up, half of the soil collected outside the *P. juliflora* canopy was mixed with the powdered *P. juliflora* leaf litter at a ratio of 60 g leaf litter/ kg soil [38].

### 4.2. Experimental Design

The study encompassed three soil treatments: soil from underneath the Prosopis canopy (UPC), soil from outside the Prosopis canopy (OPC), and soil from outside the Prosopis canopy mixed with *Prosopis* leaf litter (OPCLL). We added AC bought from Ecospot Chemicals Africa Ltd-Jacobi Aquasorb to half of the pots from each soil type at a concentration of 20 mL/L substrate [9].

Seedlings of *V. tortilis*, *B. aegyptica, Z. mauritania,* and *P. juliflora* were transplanted at the 2-leaf stage (approximately 4–10 cm tall) into pots (diameter 5 cm, height 8 cm) filled with the six soil type-AC combinations. Seedlings of each of the four tree species were grown alone (4 monocultures). In addition, seedlings of the three resident tree species were grown either with a seedling of a heterospecific or with a seedling of *P. juliflora* (6 mixtures). This resulted in 60 different soil type-AC-tree species combinations. Each soil type-AC-tree species combination was replicated 4 times, resulting in a total of 240 pots.

All pots were transferred to an empty garden bed in the nursery at the KEFRI Centre in Marigat, Kenya. The pots were arranged in a randomized block design, with each block comprising one replicate of each of the soil type-AC-tree species combinations. On the day the potting tubes were transferred to the garden bed, height was measured for all seedlings. The newly transplanted seedlings were shaded for two weeks to protect against shock and enable them to adjust to the new conditions. The pots were evenly watered twice a day, early in the morning and late in the afternoon, using a fine-nozzle watering can. After three months, all seedlings were harvested, and their survival and height were recorded.

### 4.3. Data Analysis

The statistical software R, version 4.4.2 [39], with the interface R Studio, Posit team (2024) [40], was used. The experiment included a total of 240 pots. Of these, 216 contained resident tree seedlings and were used for the regression analyses, while 24 pots contained only Prosopis seedlings and were used separately to assess the direct effect of activated carbon (AC) on seedling performance. Within the dataset of resident seedlings, three samples were excluded as outliers: two samples were excluded due to extreme baseline height values that could not be explained and that were confirmed as outliers using Rosner’s test from the package EnvStats (version 3.0.0 [41]), and one additional sample was identified as an influential data point in the model assessing height after three months, based on diagnostics from the Q–Q plot, residual–fitted comparisons, and Cook’s distance.

The final dataset for the analysis of the binary variable survival after three months thus included 213 samples. A logistic model was fitted, including soil type (UPC, OPC, and OPCLL), AC (yes, no), competition (none, resident tree species, *Prosopis*), the three two-way interactions of the aforementioned variables, height baseline, and resident species as fixed factors.

For the analysis of height after three months, all samples that were still alive at this point were considered (*N* = 129). A linear regression (factorial ANCOVA) model was fitted, including the same main effects and two-way interactions among soil type, activated carbon, and competition treatment, with baseline height included as a covariate and species as an additional fixed factor. Heteroscedasticity was detected using the Breusch-Pragan-Test from the package ‘lmtest’ (version 0.9.40) [42]; thus, heteroscedasticity-consistent standard errors were calculated, using HC3 as suggested by Hayes and Cai [43]. The direct effect of AC on *Prosopis* and on resident tree seedlings in the absence of competition was assessed using the Wilcoxon rank-sum test.

## 5. Conclusions

Our study provides evidence for allelopathic effects of *P. julilfora* on resident tree species and thereby corroborates results from other studies in which the role of allelopathy in plant invasions was assessed by combining multiple experimental techniques. Further studies are needed to better understand the allelopathic potential of *P. juliflora* against a wider range of plant species. Identification of allelopathic substances exuded by *P. juliflora* and monitoring their concentrations in the soil over time will help manage allelopathic interactions between *P. juliflora* and resident plant species in restoration projects.

## Data Availability

The original contributions presented in this study are included in the article/Supplementary Material. Further inquiries can be directed to the corresponding authors.

## Supplementary Materials

The following supporting information can be downloaded at: https://www.mdpi.com/article/10.3390/plants15040571/s1. Table S1: Results of the logistic regression on seedling survival (*N* = 213). Significant results (*p* < 0.05) are in bold.; Table S2: Results of the linear regression on seedling height (*N* = 129), computed with heteroscedasticity consistent standard errors. Significant results (*p* < 0.05) are in bold.; Figure S1a: The effect of soil type and competition on survival of resident tree species in the absence of activated carbon.; Figure S1b: The effect of soil type and competition on survival of resident tree species in the presence of activated carbon.; Figure S2a: The effect of soil type and competition on seedling height of resident tree species in the absence of activated carbon.; Figure S2b: The effect of soil type and competition on seedling height of resident tree species in the presence of activated carbon.

## Abbreviations

UPC	underneath *Prosopis juliflora* canopy
OPC	outside *Prosopis juliflora* canopy
OPCLL	outside *Prosopis juliflora* canopy mixed with leaf litter
