# Supplementary material for: Leaf Litter and Soil-Mediated Impacts of the Invasive Tree Prosopis juliflora on Seedlings of Resident Tree Species
Source: Plants (Basel). 2026 Feb 11;15(4):571. doi: 10.3390/plants15040571 (PMC12944309; doi:10.3390/plants15040571)

Table S1. Results of the logistic regression on seedling survival (N = 213). Significant results ($p < 0.05$) are in bold.

**Characteristic**		**Log (OR)**	**95% CI**	***p*-value**
(Intercept)		3.4	0.38, 6.8	0.035
Soil Type	OPC	—	—	
	UPC	-2.2	-4.4, -0.45	**0.022**
	OPCLL	-4.6	-6.9, -2.6	**<0.001**
Activated Carbon (AC)	0	—	—	
	1	0.64	-1.2, 2.6	0.5
Competition	None	—	—	
	Resident	-1.0	-3.2, 0.87	0.3
	Prosopis	-1.5	-3.7, 0.40	0.14
Survival_baseline		-0.07	-0.47, 0.33	0.7
Species	*Balanites*	—	—	
	Vachellia	-0.64	-1.5, 0.20	0.14
	Ziziphus	-1.5	-2.4, -0.68	**<0.001**
Soil Type * AC				
	UPC * 1	1.7	-0.01, 3.5	0.052
	OPCLL * 1	2.4	0.61, 4.2	**0.009**
Soil Type * Competition				
	UPC * Resident	0.57	-1.7, 3.0	0.6
	OPCLL * Resident	0.87	-1.5, 3.5	0.5
	UPC * *Prosopis*	1.0	-1.2, 3.5	0.4
	OPCLL * *Prosopis*	2.4	0.22, 4.9	**0.040**
AC * Competition	1 * Resident	-0.22	-2.2, 1.7	0.8
	1 * *Prosopis*	-0.79	-2.7, 0.97	0.4

Table S2. Results of the linear regression on seedling height (N = 129), computed with heteroscedasticity consistent standard errors. Significant results ($p < 0.05$) are in bold.

**Characteristic**		**Beta**	**95% CI**	
(Intercept)		18	13, 22	<0.001
Soil Type	OPC	—	—	
	UPC	-3.2	-4.9, -1.5	**<0.001**
	OPCLL	-8.7	-11, -6.9	**<0.001**
Activated Carbon (AC)	0	—	—	
	1	0.15	-1.9, 2.2	0.9
Competition	None	—	—	
	Resident	-3.1	-4.3, -1.9	**<0.001**
	Prosopis	-3.5	-5.5, -1.6	**<0.001**
Height_baseline		0.15	-0.49, 0.79	0.6
Species	*Balanites*	—	—	
	Vachellia	0.43	-0.53, 1.4	0.4
	Ziziphus	-0.72	-1.8, 0.33	0.2
Soil Type * AC	UPC * 1	1.9	0.12, 3.8	**0.036**
	OPCLL * 1	3.9	2.0, 5.8	**<0.001**
Soil Type * Competition	UPC * Resident	-1.6	-3.8, 0.72	0.2
	OPCLL * Resident	-2.5	-5.8, 0.87	0.15
	UPC * *Prosopis*	-2.0	-4.6, 0.70	0.15
	OPCLL * *Prosopis*	1.3	-0.95, 3.5	0.3
AC * Competition	1 * Resident	4.9	3.0, 6.9	**<0.001**
	1 * *Prosopis*	2.5	0.38, 4.7	**0.022**

Figure S1a. The effect of soil type and competition on survival of resident tree species in the absence of activated carbon.

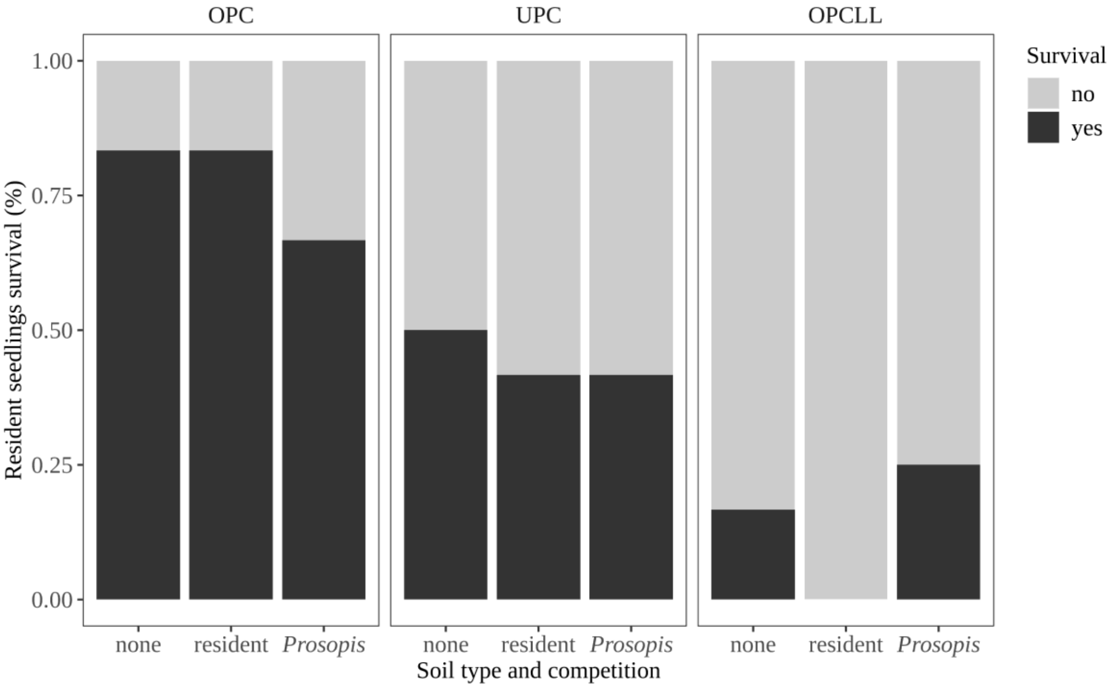

Figure S1b. The effect of soil type and competition on survival of resident tree species in the presence of activated carbon.

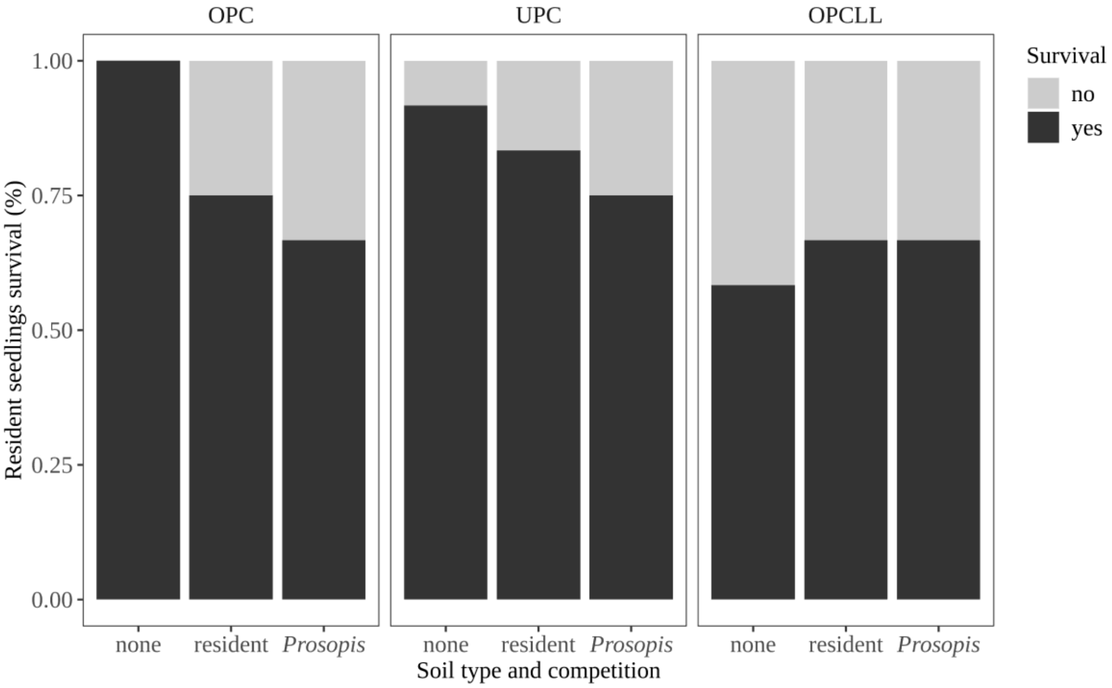

Figure S2a. The effect of soil type and competition on seedling height of resident tree species in the absence of activated carbon.

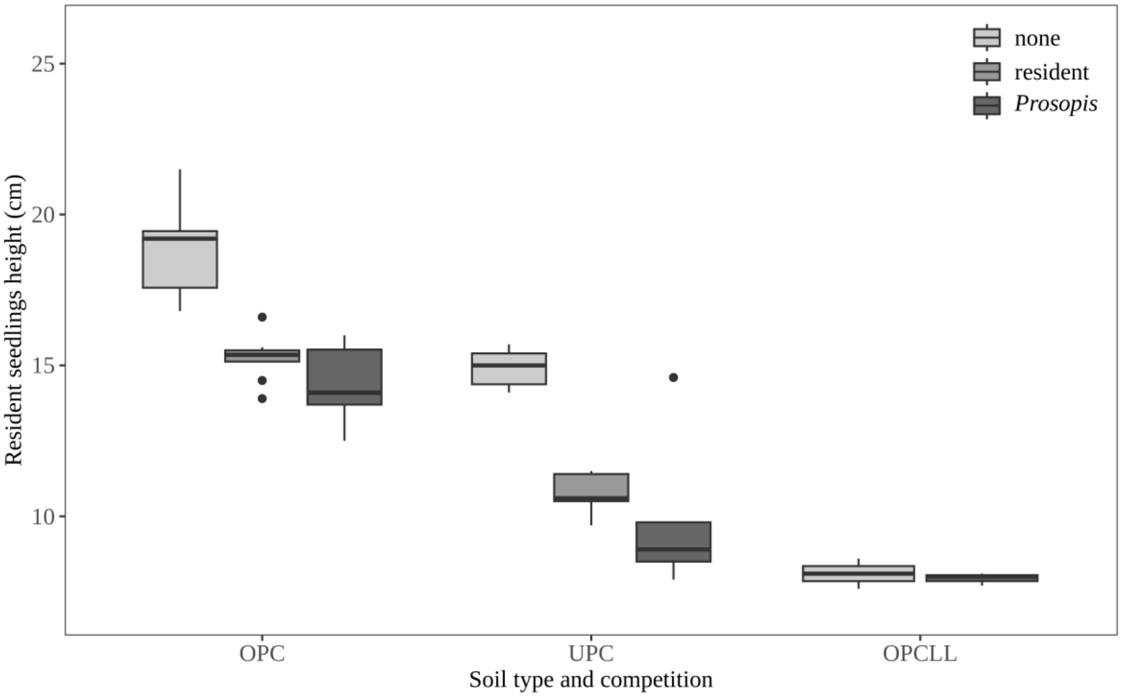

Figure S2b. The effect of soil type and competition on seedling height of resident tree species in the presence of activated carbon.

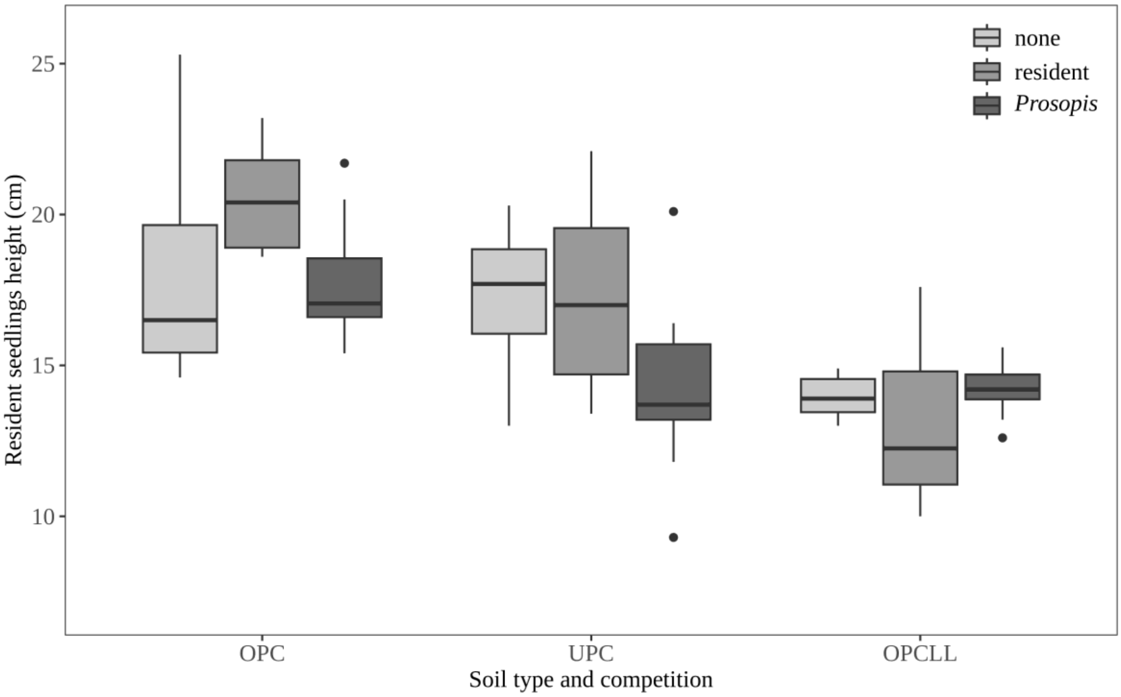

Supplement: Supplementary file 1 [file plants-15-00571-s001.zip › plants-4063871-supplementary.pdf]
